# Raising Boys for the Navy: Health, Welfare, and the British Sea Services, 1870-1905

**DOI:** 10.1093/jhmas/jraa054

**Published:** 2020-11-19

**Authors:** Elise Juzda Smith

**Affiliations:** Department of History/Centre for the History of Medicine, University of Warwick

**Keywords:** child welfare, auxology, anthropometry, degeneration, naval medicine, Royal Navy, mercantile marine

## Abstract

Efforts to improve the quality and quantity of seafarers in the Royal Navy and merchant service became a particular concern amidst the degeneration debates of late-Victorian Britain. Maritime reformers not only promoted fitness in adult sailors, but also particularly sought to improve health and physique of boy recruits in order to rear a new generation of healthy sailors. This article shows how both services experimented with tighter admission criteria and dietary and exercise reforms, and became early advocates of using metrical standards to exclude all but the fittest, healthiest boys from training opportunities. While the physical monitoring of boy recruits undoubtedly showed the value of early lifestyle interventions in fostering healthy development, the rising physical standards of British seafarers in this period was just as much the result of restrictive medical examinations as a commitment to welfare initiatives.

Boys were a familiar sight aboard British ships in the nineteenth century. The idea that sailors were best bred from a young age had been posited a century earlier, and few questioned the tradition of recruiting boys well into the steam age.^1^ No longer were those of reduced stature required to navigate the tight spaces below decks on wooden vessels, ferrying gunpowder during battles or cleaning cramped quarters.^2^ Yet decades after the golden age of sail, when the construction of massive iron ships had obviated any practical need for small hands and builds, both the British Navy and mercantile marine continued to enlist boys. Far from being desired for their diminutive size, recruiters increasingly selected candidates based on their physical vigour, their bodies scrutinized for signs that they would one day mature into robust adult seafarers.

This article examines the recruitment and training of boys for the British sea services in the late nineteenth century, showing how a concern with the quantity and quality of adult seafarers incited efforts to rear a new generation of healthy sailors. Both the Royal Navy and merchant service had experienced manning issues in mid-century that provoked considerable unease over the supply of capable candidates. Within the sweeping reforms that were enacted to increase the appeal of a maritime career, the cultivation of health and fitness amongst personnel played a prominent role. Institutions designed to transform boys into sailors, such as the Marine Society’s training ships and the Royal Navy’s Greenwich Hospital School, tightened their admission criteria and ameliorated their dietary and exercise regimes. As fears of war and invasion mounted at the close of the century, such initiatives offered a rejoinder to concerns over the availability of candidates ready and able to man the fleet.

The Navy’s particular investment in promoting optimal physical development demonstrates the specialist concerns motivating the growth of child welfare in the late nineteenth century. As historians of childhood have noted, a proliferation of advice on healthy child rearing emerged during this period and represented a distinct subfield of the public health movement.^3^ It also coincided with the rise of physical culture within society as a whole, leading to an idolization of strong, athletic male bodies that invariably informed notions of combat fitness.^4^ Yet alongside such aspirational ideals, an omnipresent fear of physical deterioration coloured perceptions of the body. Poor health outcomes, particularly amongst impoverished city dwellers, hinted at a progressive national decline that might one day render Britain incapable of defending its borders or commercial interests.^5^ This state of alarm invariably settled on the armed forces, fuelling calls for greater investments in personnel to safeguard the nation. As Heinrich Hartmann has argued, the concept of “military strength” crystallised across Europe to denote the collective evaluation of bodies and their suitability for combat in an era fuelled as much by insecurity as it was by nationalism and militarism.^6^

Unlike the British army, which recruited men aged eighteen and over, the Royal Navy was able to make use of its traditional adolescent base to defray certain elements of the degeneration panic. By fostering the healthy growth of an entire workforce from a young age, the nation was assured of a steady supply of strong, dependable, homegrown sailors. Tellingly, when the army came under fire at the turn of the twentieth century for the high level of medical rejections experienced by recruits during the Boer War, the Navy evaded similar criticism by pointing to the higher physical calibre of its crews. The benefits of raising dietary standards and promoting exercise were clear to commentators who praised the “magnificent physique” of the modern “Jack Tar.”^7^ While concerns over the weakness of the army have figured prominently in studies of the fin-de-siècle degeneration panic, the contrasting role of the Navy in cultivating a more positive image of Britain’s martial strength has received less notice.^8^ This is perhaps because the decline in the sea service was identified several decades earlier and was counteracted through a gradual process of reform. Yet the Navy’s particular focus on youth training as a corrective was innovative, and anticipated many of the recommendations that helped to improve the health of school children in the early 1900s. If their crews’ improved health alleviated some of the period’s pessimism, it did not produce a decisive example of nurture triumphing over nature. In the late nineteenth century, maritime training programmes for both services had raised their admission criteria to favour men and boys from more prosperous backgrounds. Despite contemporary adventure stories romanticising young men running away to sea, it became an increasingly unattainable prospect for all but the most physically promising candidates. At best, the Navy and Mercantile Marine developed techniques to measure and mitigate the effects of deprivation, while offering little hope to the worst afflicted.

This article contextualizes these developments by first examining the medical, moral, and nationalist arguments made for improving the quality of British seafarers in the nineteenth century. The manner in which these concerns shaped the training of boys for the Navy, particularly at the Greenwich Hospital School, is next examined, showing how measurements used to predict fitness in maturing bodies became tools of arbitration and exclusion as recruitment standards rose. The final section reveals how charitable schemes designed to produce merchant seamen similarly sought to institute reforms to promote healthy growth, becoming equally preoccupied with the size and strength of prospective candidates. The British sea services’ attempts to raise fitter recruits demonstrates a new commitment on the part of the state to invest in military efficiency through child welfare—albeit only for those exhibiting superior potential. It was a measure of this strategy’s success that young seafarers’ bodies were increasingly upheld as a sign of Britain’s physical promise in an age awash with anxieties over national decay.

## Able-Bodied Seamen

The concern with the health of boys destined for the Royal Navy arose in the wake of reforms designed to attract and retain a more respectable class of seafarers. As Mary A. Conley has shown, targeted efforts to improve standards across the service were successful in elevating not only the material conditions of life afloat, but also its public image.^9^ By the dawn of the new century, a cleaner, more disciplined service, composed of sober, pious, upright men, represented a newly positive vision of a livelihood formerly associated with strife and vice.^10^ This process of reform went hand-in-hand with new attitudes towards health and athleticism that helped to define the physical prowess that modern sailors were meant to embody.

The prioritisation of physical efficiency in the Royal Navy occurred in response to recurrent manning crises in the middle of the nineteenth century. Rapid mobilisations and demobilisations around the Crimean War and Indian Mutiny led to a chaotic and inefficient recruitment system facing constant shortages.^11^ This state of affairs ultimately led to the introduction of continuous service engagements in 1853 and the creation of the Royal Naval Reserve in 1859. While longer service agreements ensured that sailors could rely on consistent employment for at least a decade, the Reserve offered financial incentives to merchant seamen to supplement Naval crews in times of war. Changes were not only limited to terms of employment; the Admiralty also embarked on a programme of reforms to improve conditions afloat. Discipline was moderated, corporal punishment abolished, diets improved, and uniforms introduced.^12^ Explicit steps to eradicate vice and lure sailors from unwholesome habits were also taken. Ships were provided with libraries, temperance was promoted, and sporting competitions were organised both on ship and on shore. Whereas recruiters had once struggled to attract any but the most disreputable men to a low-paid life filled with depravations, the reformed Victorian Navy appealed to men of sounder character, who could look forward to a term of secure employment in a relatively wholesome shipboard environment. While the rehabilitation of the Navy’s image in this period has been well documented, the health implications of these shifts merit further scrutiny.^13^ This section shows how the cultivation of fitness in the Royal Navy transformed the composition of its crews and suggested the need for ever-earlier interventions into the shaping of sailors’ physiques.

The growing allure of a Naval career in the steam age precipitated a rise in the standard of candidates, which in turn permitted more rigorous medical criteria to be used in selecting crews. Prior to this period, the scarcity of volunteers meant that individuals were passed as fit so long as they did not manifest any obvious “Hurts or Disease.”^14^ Even so, recruiters were liable to gloss over physical malformations or potential symptoms in their desperation to supply ships with a full complement of sailors. A similarly-motivated laxity in medical inspections led to Invaliding Boards confronting cases of seafarers labouring under chronic diseases—sometimes with a deceptively “healthy aspect”—that ought to have been instantly disqualifying.^15^ By the mid-Victorian reform era, there was a growing sense that the Navy’s efficiency was being compromised by poor health, and that medical examinations needed to employ stricter criteria.

Fitness for the Victorian Navy was seen almost exclusively through the prism of physical integrity rather than size, although the height of most recruits was recorded from the 1830s as an identifying characteristic. While the British government was beginning to monitor stature in other employment contexts, particularly child factory labour, the medical implications of the naval data were not immediately seized upon.^16^ In 1841, the naval surgeon John Wilson contended that height, weight, and age merited greater consideration when selecting men for the service. He recommended that sailors be aged between 20 and 40, and of “middle stature” (between 5’4” and 5’8”), given that excess height was disadvantageous on small ships while men of “small frame” were often useless on larger ones, being “too light, or too much under-sized” to perform heavy duties.^17^ This reference to the inadequacy of some British seafarers was shared by many of Wilson’s colleagues, who agreed that more attention ought to be paid to the physical condition of recruits.^18^ A careful search for signs of internal disorders, particularly in the heart and lungs, was seen as a necessary addition to the standard superficial examination. The Admiralty formally responded to such critiques by revamping their guidance for medical examinations in 1862, issuing an extensive list of disqualifying traits that included skin complaints, asthma, defective vision, and disordered intellect—all deemed signs of a “feeble constitution” that might impair efficiency.^19^

By the dawn of the steam age, the dictum “health is power” had come to offer a justification for a concentrated investment in hygiene reforms to reduce outbreaks of disease.^20^ Although improving the sanitation and ventilation of vessels was the lynchpin of this strategy, it also incorporated efforts at modifying unhealthy behaviours. Drunkenness, dirtiness, imperfect discipline, overexertion, and the solicitation of prostitutes ashore—all were habits deemed ruinous to health. They were also thought to be integral to the character of seafarers, which was often patronizingly described by reformers as rash, childlike, and careless.^21^ Correcting the archetypal Bluejacket’s perceived flaws and redeeming his image was a project that absorbed the Admiralty for much of the nineteenth century.

Two behavioural problems that particularly attracted the attention of the Medical Department were alcoholism and the high incidence of venereal diseases. Despite the long association of both conditions with masculine indulgences in the Navy, they were also directly correlated with mental, moral, and physical degeneration in the Victorian era.^22^ Given the pervasiveness of venereal diseases in the armed forces, the introduction of successive Contagious Diseases Acts (1864, 1866, 1869) was welcomed by medical officers. Historians such as Judith Walkowitz have highlighted the discriminatory bent of these acts in their policing of prostitutes, a gendered focus that provoked outrage amongst nascent campaigners for women’s rights.^23^ Yet given their myopic concern over losses to the service (with 280 sailors being stricken daily), the Naval Medical Department was persuaded that rates of infection were “much checked” by the legislation’s passage.^24^ Temporary sickness was not the only effect of venereal diseases that concerned medical commentators; the potential for permanent damage to the constitution being transmitted to future generations loomed large. One surgeon warned that the spread of syphilis throughout Britain’s seafaring families would confront recruiters who would one day be scavenging amongst the “scanty and sickly offspring of a tainted race.”^25^ The large number of seafarers’ children unable to meet the Greenwich Hospital School’s fitness requirements later in the century bolstered such fears and led to a redoubling of efforts to curb the sexual indiscretions of their fathers.

The propensity towards drink in the service, meanwhile, incensed surgeons who believed it weakened the constitution and rendered men more vulnerable to every form of sickness, from bilious complaints to gout, epilepsy, and ulcers.^26^ The manner in which alcohol consumption was tied to manliness exacerbated the problem—the naval hygienist John MacDonald described how a newly-raised lad would be “ridiculed as a milksop” if he refused his half-pint of spirits, but be praised for his “manly and seamanlike qualifications if he drank it with avidity.”^27^ Convincing boys not to over-indulge became an objective of many of the preliminary training schemes for the Navy, with dedicated lantern shows projecting the role of “strong character” in resisting the dangers of drink.^28^ Although naval medical commentators had long observed the physiological damage incurred by intemperance, their belief in its hereditary consequences stoked broader fears of progressive decline. Such concerns were explicitly racialised and anticipated eugenic fears of inter-generational decay. For the surgeon Alexander Bryson (later to become Director of the Naval Medical Department), the tendency to drink was a “degrading propensity” that was sinking “the white man in the scale of reason below the savage of the bush.”^29^ This spectre of national devolution was a powerful spur to action, and the Admiralty appointed a Grog Committee in 1850 to reassess the drink allowance in the service.^30^ As a consequence, the rum ration was halved for most ranks, and denied completely to cadets and second-class boys. While drinking culture remained part and parcel of naval life in the nineteenth century, the particular damage it posed to the young was inherent in the move towards prohibition for boy recruits. The temperance movement also gained traction amongst older members of the Navy, aided by certain captains’ active endorsement of “taking the pledge” and their recommendation of temperance hotels. In 1879, the Royal Navy Temperance Society claimed to have convinced seven to eight thousand “pledged abstainers” out of the Navy’s complement of almost fifty-eight thousand men: a proportion considerably higher than they achieved ashore despite the Navy’s reputation for inebriety.^31^

The new emphasis on clean living similarly encouraged the rise of dedicated exercise programmes and sporting competition for naval personnel. Such activities reflected the spread of physical culture and Muscular Christianity in late-Victorian Britain, which enforced a link between military prowess and an overtly athletic expression of masculinity. They were equally designed to compensate for the technological changes that were transforming life afloat. While the fitness of seafarers had been taken for granted on wooden ships given the physical effort required to manage the sails, the adoption of steam propulsion was presumed to encourage inertia. In 1897, staff-surgeon C. March Beadnell measured the crew of *HMS Powerful* and suggested that sailors’ bodies were undergoing a process akin to evolutionary change. Aboard steamers, the introduction of intricate machinery had led to a “critical phase” of adaptation, where the intellect was overtaking the “*corpus virile*” in importance as the role of manual labour declined.^32^ Not all commentators feared such changes, however; the naval captain Alfred Jephson wrote in 1901 that the modern sailor was both possessed of higher mental faculties and a “broader… thick-set” build which substituted strength for dexterity.^33^ If sailors no longer got their traditional exercise in the rigging, muscle was still needed to manage the weight of heavy guns and machinery.

Sporting matches, drill exercises, and gymnastic displays were also actively promoted by the Navy to assuage those fearful of a decline in physical standards. While technology may have rendered excess strength superfluous, the athleticism of naval crews was as visible a manifestation of Britain’s maritime supremacy as its investment in ever larger and more elaborate ships. Promotional images of the late-Victorian sea service proudly depicted sailors engaged in rowing, running, football, cricket, or the tug-of-war. Such wholesome entertainments equally emphasised the Navy’s newfound respectability. The Royal Naval Exhibition, held in London for six months in 1891, featured a full programme of drill routines and athletic performances, where health, patriotism, and valour radiated from the physiques on display.^34^ Even the sailors’ posture was improved: instructions in gymnastics endowed them with an “upright carriage, firm swinging step, and generally smart appearance.”^35^

For all the optimism of such exercises, however, doubts were continuously raised over the quality and quantity of British seafarers in the late nineteenth century. The vast expansion of shipbuilding in this period meant that the Navy’s demand for sailors often exceeded supply, and a complete wartime mobilization threatened a deficit in the thousands.^36^ The merchant service, meanwhile, was in no position to make up the shortfall—in 1873 the Royal Commission on Unseaworthy Ships heard countless witnesses complain of dirty and disorderly vessels dependent on imported labour.^37^ While underscoring the need for more British sailors, these revelations equally fuelled a xenophobic distrust of foreign sailors—particularly Lascars, Scandinavians, and the Irish—who had long been blamed for “introducing laziness and filthy habits” into British ships.^38^

The biased discourse around seafarers’ character from the mid-century onwards suggested that the Navy’s manning issues were not only a matter of finding enough men to serve, but of inducing the *right kind* of man to do so. As this section has shown, such candidates were increasingly envisaged as not only being fit and healthy, but also British, literate, pious, abstentious, and generally “respectable”—with all the prejudices such a description entailed. The next section examines how the Admiralty sought to resolve the dilemma of attracting healthy, wholesome recruits by literally raising a fighting force from boyhood into maturity. The Navy’s plan to engineer a superior breed of sailor was particularly enacted at the Greenwich Hospital School, which was transformed into a testing ground for new theories surrounding children’s health, growth, and physical fitness, and which pioneered new methods of measuring these qualities in a bid to identify the service’s likeliest prospects.

## The Cradle of the Navy

In 1882, the Admiralty placed a notice in several newspapers stating that “a large number of healthy, respectable boys” was required for the Royal Navy, and that the qualifying measurements, in height and chest girth, had been lowered to attract more candidates.^39^ In response, the satirical periodical *Judy* produced a poem ridiculing the notion of “dear little brats” replacing “seamen bluff” accompanied by a sketch of motley youth smiling vapidly and picking their noses.^40^ The image may have been absurd, but the Navy’s appeal reflected a long-standing policy of admitting adolescent boys into its ranks. Prior to obtaining a contract aboard a ship, young men were expected to complete a one-and-a-half year course aboard a training ship, starting when they were 15 or 16. Boys of an even younger age were eligible for naval instruction at the Admiralty-run Greenwich Hospital Schools (GHS), which admitted candidates as young as 11. The search for “healthy, respectable boys” for such schemes demonstrated the Navy’s desire to rear sailors already endowed with the desirable attributes it was inculcating in its adult crews. That it occasionally had to lower its entrance requirements—as exemplified in its 1882 advertisement—shows that the manning needs of the service necessitated a flexible conception of fitness for its youngest recruits.

Yet given the variability of its boy candidate pool, the Royal Navy engaged with ideas around fostering healthy growth through dietary and exercise provisions. Their trials and errors in this pursuit offer an early example of managing and monitoring children’s welfare through a complete control of living conditions. Unlike other state institutions for children, such as workhouses or orphanages, the Navy’s training schemes had an obligation to optimize physical strength rather than provide mere sustenance. To this end, they became enthusiastic adopters of using measurements as a determinant of health and fitness, and grappled with questions around standardization, methodology, and the consistency of growth rates. The constant need to revisit these issues and revise their approach reflected broader uncertainties about the reliability of such techniques, and whether nurture could ever make up for nature’s deficits when it came to shaping physiques. Despite frequent setbacks, by the close of the nineteenth century the Navy had successfully used its training programmes to raise the standard of its recruits. This was accomplished in part by changes to the admission criteria, but also by monitoring the physical effects of exercise and dietary reforms. It was through these efforts that the Royal Navy helped to establish the importance of environmental factors in raising healthy children. It is no coincidence that the Greenwich Hospital School became a vehicle for the Navy’s ambitions in this regard; the vast expansion of elementary education in the late nineteenth century offered an opportunity to monitor children’s welfare in an unprecedented manner. As a state-run, charitable school with a mission to prepare boys for maritime careers, the GHS long struggled with limited funds and vision before aspiring to raise its standards to match those of elite public schools. While the Navy’s innovative educational policies at the GHS have received some historical attention, this section particularly looks at how the school’s administrators embraced anthropometric methods to evaluate and improve the fitness of their charges.^41^

The Greenwich Hospital School, which opened its doors in 1712, was always under the Admiralty’s jurisdiction, although it was not initially intended as a training ground for the Royal Navy. Rather, its mission was charitable; the school’s Royal Charter stipulated its intention to educate the children of slain or disabled seamen. A century later, the establishment of the Royal Naval Asylum (1809) freed the GHS from its responsibility for naval orphans, allowing it to expand beyond its philanthropic remit. While the functions of the Asylum and Hospital School were shortly merged again in 1821, its charitable and scholastic ambitions were kept distinct. The Lower School was henceforth responsible for supporting younger, destitute children, while the Upper School educated older boys whose admission was opened to a variety of schemes, including patronage.^42^ Increasingly, both schools were seen as a fertile training ground for the Royal Navy. Girls ceased to be admitted to the Lower School in 1841, and nautical instruction became a focus of the curriculum at both levels. As per its name, the School was directly administered by Greenwich Hospital, an adjacent home for retired sailors governed by the Admiralty.

The Admiralty became particularly concerned with improving the status of the Upper School following the 1821 merger and reshaping it into a more selective institution. Admissions were dependent on showing basic literacy and numeracy, and applicants needed to have their “fitness”—nebulously defined—approved by the Captain and Headmaster, with all candidates subject to an interview with the School’s Governing Board. Alongside academic reforms, the school’s dietary was gradually increased, and funds were allotted for the purchase of exercise equipment. Raising the standards of the Upper School was as much directed towards improving the pupil’s bodies as their minds. Indeed, the necessity of superior strength and size were made clear when four graduates sent to serve aboard the *HMS Madagascar* in 1828 were rejected as being “too small” for the Royal Navy—an embarrassment that led the school to assess the pupils’ fitness before they were discharged.^43^ Despite the lack of formal height standards for the service at this stage, a correlation between larger size and suitability for a naval career was unofficially enforced as manning needs contracted in the early years of the Pax Britannica. Indeed, throughout the nineteenth century the emphasis on fitness as a recruitment criterion proved somewhat fickle depending on manning requirements.

As noted in the last section, mid-century naval reforms had prioritized sailors’ health in a bid for greater efficiency and improved decorum. As medical inspections became more thorough across the sea service, the desire for bigger, healthier bodies invariably trickled down to its training schemes. At the GHS, the Upper School began explicitly targeting boys from more prosperous backgrounds. By the 1850s it had a complement of 400 students, a quarter of which comprised officers’ sons, with the remainder being the offspring of either marines or naval and merchant seamen.^44^ In order to limit registration to “respectable” boys, copies of the parents’ marriage certificate and a record of the applicant’s baptism were requested. A certificate declaring the boy “a proper object for the Charity” was also required, but the selection process was unquestionably governed by middle-class morality. Applications were routinely rejected for boys born out of wedlock, and school officials ruthlessly pursued cases with suspicious paperwork, threatening desperate mothers with exposure and prosecution for falsifying dates or marital status.^45^ Optimal health similarly became a marker of quality. Parents and guardians were warned that it would be “perfectly useless” to send children with any “ringworm, soreness of head” or showing signs of temporary sickness.^46^ Even at the Lower School, where orphaned and destitute children were still admitted, there was a strict expectation of physical and mental ability, with forms declaring in bold red ink that boys “must be free from any impediment of speech, or other infirmity of body or mind.”^47^

If poor health was a barrier to admission to naval training in the mid-nineteenth century, it was traditionally qualified through symptoms or a sickly appearance. The Admiralty’s decision in 1831 to set minimum height standards for naval recruits introduced an alternative, quantitative means of gauging fitness: candidates aged fourteen and above now had to be at least 4’9” tall to join a ship as a second class boy.^48^ While this move coincided with the practice of documenting heights on service records more broadly, there was no rationale provided for the size limit that was set. Given the dearth of contemporary auxological knowledge, it was perhaps unexpected to mandate a specific height for boys whose growth was incomplete. Rather than explicitly linking health and height, the minimum measurement instead codified the sense that smaller bodies were unsuited to life afloat and established a threshold at which a boy’s size was no longer deemed a liability. It is unclear how ambitious the Navy’s height target was given the lack of comparable data from the general population—it was, however, an inch taller than the average male factory worker of the same age, in an era when their diminutive size was provoking concern about the effects of excess labour and urban poverty.^49^ The Navy’s ability to restrict recruitment to slightly larger boys reflected a manning situation that was far from desperate, and anticipated the construction of larger vessels as naval architecture progressed.

If the Admiralty’s initial imposition of a height standard suggested a growing consensus over what size a sailor *should* be, their expectations were periodically recalibrated. In 1841, the standard for 14-year-olds was raised an inch to 4’10”, and a weight requirement of 95lbs. was added.^50^ By 1851, reports that this standard was “impractical” led to a reduction in the minimum height to 4’8” and the weight limit was jettisoned—although officers were strictly cautioned to select boys who were “well grown.”^51^ Yet as the Manning Committee was informed following year, this loosening of standards rapidly led to the admission of boys who did not “afford a fair prospect of attaining a proper Stature in after Years.”^52^ The height standard of 4’8” for 14-year-olds was firmly reasserted, without exception. The Committee agreed not to reimpose a minimum weight for boys, reasoning that bulk could always be assured with ample feeding—whereas gains in height seemingly defied prediction. Although early anthropometric studies showed that boys often experienced a growth spurt between the ages of 13 and 15, it was anecdotal evidence—rather than data—that informed the decision to retain a fixed standard for this volatile age group.^53^

The 1852 Manning Committee’s consideration of recruits’ physique particularly led them to scrutinize GHS graduates, whose size and strength were disparaged by training ship captains who preferred larger boys recruited directly from maritime families.^54^ Lieutenant Rouse, one of the school’s superintendents, testified to the boys’ athleticism, but confirmed that applicants were not excluded on the basis of size—unless they were particularly “dwarfish.”^55^ Given the students’ consistent inability to qualify for the Royal Navy over the next decade, the GHS finally established minimum measurements for applicants in 1871. Yet the ostensible authority of numbers in this period did not allow for simple resolutions. There was constant debate about which dimensions should be recorded, the target sizes to be set for different ages, and the consequences for boys who failed to meet the criteria during their training. The lack of data about growth equally hampered efforts to set standards for 11 or 13-year-olds to ensure they attained the Navy’s height and chest requirements by age 15. Despite the sporadic pursuit of anthropometric surveying in mid-Victorian Britain, the military establishment acknowledged the discipline’s potential to guide such decisions. An 1862 lecture by William Aitken at the Army Medical School in Chatham cited a range of numerical data culled from the Statistical Society to designate healthy heights for every age bracket, and it is likely that similar data informed the Navy’s admission standards.^56^

At the GHS, however, continuous objections and revisions to the measurements for entry into the Upper School showed that numerical barriers could be surprisingly porous. The 1871 admission criteria initially required 13-year-olds to be 4’6” in height, 25 inches around the chest, and to weigh at least 72lbs.^57^ Around this time, the Navy was admitting candidates at the age of 15, who were at least 4’10.5” tall with a 29-inch chest, so steady gains needed to be made at the school.^58^ The GHS Medical officers were nonetheless given leeway to admit undersized boys as long as they gave promise “ultimately of good physical development.”^59^ This discretionary clause, however, soon led to an influx of smaller boys, and also resulted in candidates arriving with poor vision or a “deformity of the chest.”^60^ This prompted the school’s headmaster, Charles Burney, to write to the Lords of the Admiralty requesting that no further exceptions be made, as such students invariably failed to meet the Navy’s standards.^61^ Equally, a plea was put in for the chest circumference to be raised to a 26-inch minimum for 13-year-olds, to offer better odds of expanding the required three inches by age 15.^62^ The question of how much a boy could grow within these two decisive years steered the Royal Navy’s engagement with anthropometry and growth research, although very few points of agreement emerged. The Admiralty’s flexibility when it came to the GHS standards suggested they saw growth as more unpredictable than the school’s officials did—certainly when it came to growth spurts before the age of 15, when their rigid requirements for training ship admittance were enforced. Underlying this ambiguity was a calculation of risk: how much time and money might be spent educating boys who would potentially be unfit for the sea service? Despite their ultimate jurisdiction over the GHS, the Lords of the Admiralty were more prepared to gamble than the school’s administrators—either owing to a genuine belief in the precariousness of growth rates, or because they had no direct relationship with the boys whose futures were being decided.

The GHS problem with adopting measurements as an arbitrator of fitness in the late nineteenth century equally reflected the general lack of methodological standardization that accompanied anthropometric surveying. Although stature was easily determined with a measuring stick or tape, weight proved more challenging to record given the imprecision of contemporary scales.^63^ Chest measurements were even more contentious, despite the Navy imposing a minimal circumference in 1873 following a longstanding preference for broad-chested recruits—narrowness ostensibly being a sign of constitutional weakness.^64^ Problematically, however, results taken off the same candidate could vary depending on where the tap was placed around the upper body, leading to inconsistencies that were exacerbated by the manner in which chest size varied between an inhalation and exhalation.^65^ A strict procedure was needed to produce a universal standard: chest girth was best recorded with the boy standing with hands above his head, backs together, counting to 30 to 40 “in a steady way.”^66^ Such care was evidently not always taken, as several GHS boys were rejected by the examining officers of the training ships they were sent to despite passing the school’s final medical inspections. When the *HMS Excellent* deemed one GHS boy an inch too short around the chest in 1869, the school’s principal, Robert Holme, objected that “special pains” had been taken to ensure accuracy, and that the relevant measuring tape was closely examined and “appeared to be correct.”^67^ When the same problem arose with a different boy several months later, Holme wrote the Captain of the *Excellent* suggesting potential inconsistencies in their measuring practices. The GHS, he noted, always took “the girth of the chest just below the nipples when the arms [are] raised above the head,” and asked whether the same method was followed on the ship. Lifting the arms above the head during the examination, he noted, produced a higher chest circumference than when the measurement was taken with arms held down.^68^ The lack of uniformity in measuring procedures clearly hampered efforts to strictly enforce the same standards across the service. It may equally have been the case of misaligned medical cultures; some medical officers aboard training ships were fleet surgeons with direct experience of working with adult naval crews, while the GHS employed civil medical officers. George William Armstrong, who was the chief medical officer of the school throughout the 1870s and 1880s, had a degree of expertise in children’s health from his prior position at the Central London District School at Hanwell.^69^

If the GHS was carefully honing its measurement practices throughout the 1860s and 1870s, it was far from seeing improvements to the students’ physiques. Indeed, an attempt in this period to remodel the school after pauper institutions that emphasized manufacturing skills over nautical preparations (justified by the boys’ low qualification rate for the Navy) meant pupils now spent long hours toiling in workshops on a reduced diet. This industrial system of training had been introduced following an investigation by the Admiralty in 1870 that aimed to reduce the school’s expenditures, which were ultimately supplied by parliament through the Exchequer.^70^ The savings had their own cost, however; health outcomes plummeted during this “lowest and unhappiest period” in the school’s history.^71^ GHS graduates became so physically ill-adapted to a life at sea that the Admiralty commissioned a new investigation of the school in 1881 to determine whether the accession rate into the Navy could be increased. This committee’s emphasis on lifestyle and dietary reforms demonstrated a new willingness to consider factors beyond qualifying measurements in shaping healthy bodies for the Navy, and a commitment to invest the funds necessary to fulfill the school’s original purpose. The rising prominence of military statistics in shaping policy still ensured that there would be a role for anthropometric data, however; the efficacy of the various measures being trialed would be gauged in gains to the boys’ height and weight.

The Admiralty’s 1881 investigative committee principally attributed the GHS students’ “general failure of development and robustness” to dietary changes enacted in 1876 which had substituted a large portion of the meat allowance for additional grains—another cost-cutting recommendation that backfired.^72^ Students had been deprived of “flesh-forming” nutrients which led to stunted growth. Prior to the new victualling system being introduced, the boys had been of a similar height and weight to those in public schools; five years later they lagged far behind.^73^ When these findings were published, the medical journal *The Lancet* lashed out at the decision to reduce the quantity of meat by 12 ounces weekly, leaving the boys in a state of “semi-starvation.”^74^ This negative publicity spurred the GHS administrators to augment the meat ration and introduce more fresh produce into the diet, not only to raise the height of their charges, but also to salvage their own reputation. Moreover, they sought to provide confirmation of the experiment’s success through numerical means: the boys were “carefully weighed and measured” when the new dietary was first introduced, and again after six months. The results showed steady increases in the boys’ size and directly correlated accelerated growth with dietary reforms.^75^ Qualitative improvements could also be observed; the school’s medical officer reported that the boys looked “stouter, stronger and healthier” with “very obvious” improvements to their appearance.^76^ Yuriko Akiyama has pointed to how these trials were directly tied to the strength of the Navy, and the military efficiency of the nation at large.^77^ They also offer a clear demonstration of how physical measurements were being used not only as a recruitment criteria, but also as a means of monitoring the medical effects of children’s welfare reforms. Through its investment in the GHS boys’ long-term prospects, the Navy found itself at the vanguard of such calculations.

Although the industrial training system at the school was abandoned in favour of restored system of nautical preparations, including an emphasis on strength and fitness, the Admiralty deemed it prudent to institute yearly inspections of the boys’ health.^78^ These commenced in 1884 and were undertaken by a select group representing a range of naval, medical, and educational expertise: the President of the Royal Naval College, the Captain of Training Ships, the Director General of the Medical Department, the Inspector of Naval Schools, and the Superintendent of the Greenwich Hospital Branch. Their first tour of the GHS yielded a vision of “clean, cheerful and healthy” boys, although several who had been “admitted as cases of charity” had physiques that were “decidedly below the average.”^79^ However, the committee was satisfied with the overall welfare of the boys. The head of the Medical Department, J. W. Reid, credited the “marked improvement” since 1881 to the students’ more liberal diet.^80^ Quantitative data was once again harnessed in support of such claims: charts showing increases to the boys’ heights and weights were appended to the report. The idea that health and fitness were contingent upon dietary provisions and physical training became a favoured theme of the inspectors in subsequent years. They advocated greater spending on exercise equipment, for example, and warned against the muscular damage incurred by carrying heavy loads.^81^ Their emphasis on dietary improvements meant the school now adjusted their menus to ensure that dishes for which there was a “rooted dislike” (such as boiled mutton) were replaced by more appetizing options.^82^ By the late 1890s, the school had so completely jettisoned its former reputation for stinginess that its meals were advertised as “excellent in quality and abundant in quantity.”^83^ Tantalizing accounts of the “festival pudding,” shared amongst the students thrice yearly, suggested no expense was being spared; the massive dessert incorporated 50 lbs. of sugar and 180 eggs. This largesse continued aboard the Navy’s training ships in Portsmouth and Plymouth, where boys aged between 15 and 18 received a diet more generous than that allotted to regular servicemen, to better spur their growth.^84^ The effects of extra portions bread, potatoes, treacle, and salt pork was apparently so noticeable that the boys’ robust appearance purportedly made them “the very best recruiter[s] for the Navy that can be got.”^85^

The newfound success of the GHS in enhancing physique was portrayed in equally flattering terms in the popular press. Pictures of its playing fields, gymnasium, and swimming pool were regularly featured in boys’ papers, military periodicals, and illustrated weeklies.^86^ By the close of the century, exercise was a fixture of the boys’ routine, particularly aboard a model sailing ship built on the school grounds, where they were taught to climb the masts and rigging—not entirely anachronistically as hybrid ships were employed well into the twentieth century.^87^ Students also participated in sports such as rugby, cricket, football, and competitions in athletics, gymnastics, and fencing. Muscular development was also fostered through drill and calisthenics, with dumb-bell exercises calibrated for different age groups.^88^ These activities formed part of the youth fitness training that from the era’s commitment to physical culture. Arising in response to the late-Victorian degeneration panic, this movement attempted to counteract poor health standards through active lifestyle interventions.^89^ Far from believing that racial decline was inevitable, its advocates championed nurture over nature, insisting that a superior physique could be cultivated through a proper diet and exercise regime. The Navy’s adoption of this approach reflected a similar optimism about the improvability of boys’ bodies through targeted interventions.

Gymnastics was seen as particularly beneficial for the young: the Navy’s training manual insisted that a “wide, healthy chest and straight spine” could only be achieved through dedicated childhood maneuvers, while idleness in these years would lead to a poor physique, “organic weakness,” and an enduring “lack of efficiency.”^90^ Drill was similarly favoured for children; it inculcated discipline and coordination, and its military associations were appealing for a nation striving to retain its martial might. From the 1870s onwards, schools across the country adopted Swedish gymnastics and drill training in a bid to develop strong and healthy bodies capable of defending Britain and consolidating its imperial gains.^91^ This militarization of children’s fitness perfectly aligned with the GHS priorities, and images of its students participating drill routines with swords and rifles exemplified its role in creating a healthy breed of future sailors.^92^

The GHS promotion of youth fitness not only expressed a new national optimism about the perfectibility of boys’ bodies, but was also a mechanism for raising the school’s standing in the 1880s and 1890s. With its sweeping programme of reforms, the GHS administrators actively sought to match the educational and health standards of the country’s leading public schools, and managed to attain the highest physical standard amongst government-run schools by the turn of the century.^93^ The creation of district school boards in 1870 had provided access to elementary education across the country, and presented opportunities for the widespread monitoring of children’s wellbeing.^94^ These board schools, however, offered only minimal provisions for diet and exercise until the Liberal reforms of the early 1900s. By contrast, the welfare of GHS students was scrupulously attended to in the late Victorian era—when concerns were raised about their eyesight, for example (a prime cause of rejection by the Navy), every effort was made to improve their “optical conditions” through sufficient lighting and reading arrangements.^95^ Anthropometric evaluations equally began to gain traction in the private sphere, as schools such as Eton recorded their pupils’ heights, weights, and girths to ensure their advantageous development.^96^ In an era when most state schools and charitable institutions were contending with serious instances of hunger and disease, the GHS was competing to match the superior standards set by elite schools and optimize growth. By the time the Education Department assumed control of the GHS in 1895, its naval administrators vociferously argued that the boys’ recreation hours—deemed essential for their physical development—should not be lost in the implementation of a standard curriculum.^97^ Because the GHS boys were uniquely destined for a naval career, there could be no question of prioritizing rote mental pursuits over physical fitness; a commitment to raising and maintaining the boys’ health remained central to the school’s management in ensuing decades.

For the all the rhetoric of helping to realize their recruits’ physical potential through diet and exercise, the Navy’s size requirement for entry into both the GHS and their training programmes remained all but unattainable for boys from disadvantaged backgrounds. While recognizing the positive effects of lifestyle interventions in shaping larger and stronger bodies, their strategy was primarily directed at maximizing the physiques of larger boys rather than building up the undersized and disadvantaged. Indeed, while standards were occasionally lowered to accommodate manning needs, the difficulty of passing the Naval medical examination in the late nineteenth century remained notorious. The desire to recruit “healthy, respectable boys” cited at the start of this section was ensured not only through height, weight, and chest standards, but also a minute examination of the skin, joints, chest, teeth and eyes that would have excluded children exhibiting any physical manifestation of malnutrition and deprivation. Naval service was not for the “waifs and strays” of society, as one officer noted, but a destination for the more privileged—the sons of “small farmers, shopkeepers and artizans, who have been fed fairly, and have, therefore, some constitution on which to work.”^98^ As only a third of all applicants were able to pass the strict medical examination, it was clear that the Royal Navy was selectively refashioning its crews according to contemporary physical ideals, rather than fostering the potential of those who fell beneath them.^99^ The sea service undoubtedly strove to raise the fitness of boys in their care, and pioneered the use of measurements to assess their efforts. What it did not do was suggest that every boy was capable of being raised to their exacting standards, had they not already reached a decent size by age 13. The Navy had little to say on whether even earlier interventions might have guaranteed this growth, and thus offered scant comfort when it came to the broader, national debate on inter-generational decline. Instead, they demonstrated that it was possible to improve the physical caliber of men in their sector through a combination of selective recruitment and proper attention to food and exercise during adolescence.

Such was the competition to enter into the Navy in the late nineteenth century that one former naval surgeon recommended that boys attend a charitable or fee-based institutions designed to prepare them for the service, where their physical attributes could be provisionally assessed.^100^ As the next section shows, these independent training programmes readily absorbed the Royal Navy’s conception of health and growth as measurable phenomena. Certainly, the GHS provided an example for establishing recruitment standards and instituting dietary and exercise measures with a verifiable effect upon development; its graduates were described as the “pick and flower” of the nation’s youth by the century’s close.^101^ It particularly remained to be seen whether such strategies were transferable to training schemes for the merchant service, whose unreformed character was frequently linked to Britain’s imperiled maritime supremacy.

## No Place for Weaklings

While Victorian naval reforms were sufficiently successful that the service was increasingly seen as a respectable career prospect for healthy and hearty young men, the mercantile marine retained a reputation for moral and physical laxity. For George C. Thomas, who launched a training scheme for merchant seamen in 1903, its crews were “depraved, undisciplined, untrained […] demoralized [and] unmanageable,” or simply “the scum of the Earth.”^102^ The under-regulation of merchant shipping meant that its hygienic standards dipped far below the Royal Navy’s, provoking censure from medical commentators who saw a correlation between the filthy vessels and the condition of the men who manned them.^103^ Countless witnesses testified to the steady “deterioration” of merchant sailors before the Royal Commission on Unseaworthy Ships, which investigated the state of the service in 1874.^104^ When the Commission’s final report identified a high proportion of foreign nationals serving on merchant vessels (constituting as much as 70% of some crews), it troubled those who felt that the reserve of British sailors was not only insufficient for wartime needs, but also that the commercial sector would be left dependent on a foreign workforce of uncertain loyalty.^105^ For the protectionist commentators of the Navy League, this suggested a manning crisis by proxy, where the slogan “British sailors for British ships” expressed the conviction that the nation’s security and prosperity depended on the cultivation of new native seafarers.^106^

The response to these real and imagined anxieties was to follow the path pursued by the Royal Navy by expanding youth training opportunities for the mercantile marine and emphasizing physical fitness as both an entry criteria and principle of organization. These ideas were not new to merchant shipping; as with the Navy, the belief that a sailor’s ideal physique was best molded in youth, to properly shape the muscles, was widespread under sail. By the mid-nineteenth century, the origins of children entering the service increasingly came under scrutiny. One tract noted that crews “reared up from the offspring of half-starved paupers” would be no match for robust foreign sailors, and that Britain’s maritime competitiveness necessitated a better class of boys entering into both sea services.^107^ The idea that the merchant service was disproportionately saddled with the “scum of the parish” and required healthy, literate boys “of good character” just as the Navy did, became a recurrent theme in the manning literature.^108^ This was arguably the legacy of charitable training schemes for the merchant service, particularly those run by the Marine Society, which had been preparing orphaned and unemployed boys for maritime careers since the mid-eighteenth century. Equally, the creation of industrial training ships and floating reformatories from repurposed prison hulks had led to a degraded pool of candidates. In such conditions criminal and vagabond boys were court-ordered to learn the rudiments of seamanship, but the results were more often mutiny and scandal than the creation of healthy merchant seamen.^109^

By the late nineteenth century, a concentrated effort to improve the quality and quantity of training programmes for the mercantile marine had encouraged a greater emphasis on the welfare of boy recruits. In part, this strategy was meant to redress British ship-owners’ apparent preference for foreign seafarers over their coarse native counterparts. Although there was never an actual shortage of sailors to man the merchant fleet, xenophobic sentiments concocted a crisis over the number of British seafarers in the commercial sector and their eligibility for the Naval Reserve. The 1875 Royal Commission on Unseaworthy Ships precipitated a flurry of debate over how to raise the standing of indigenous merchant seamen. Their reputed “deterioration” was just as likely to be attributed to poor wages and inadequate sanitary measures as it was to urban and hereditary decay, but few denied that their physical and moral qualities could be ameliorated.^110^ If any professional group embodied the quagmire of degeneracy, with its taint of dirt, drunkenness, and venereal disease, it was merchant seamen.

An increase in the number of training ships, such as those already operated by the Royal Navy and Marine Society, was cited as the most obvious means of intensifying the nation’s production of healthy, wholesome sailors. Yet as the Liberal MP Thomas Brassey noted, it would be impossible to raise the deplorable character of merchant seamen unless recruiters drew from “pure and untainted sources,” such as the artisan class.^111^ He disdained ongoing efforts to train “paupers and criminals”—although other maritime reformers suggested that industrial, reformatory, and charitable training schemes could achieve a superior standard of graduate if they were better supported by the state or private subscription.^112^ In such discussions, the proliferation of healthy young sailors was deemed essential to the country’s continued dominance of global trade; the multitude of British-owned ships manned by international crews was evidently not quite British enough.

The patriotic promotion of healthy, young British seafarers was most energetically promoted by the Navy League, an organization established in 1895 to lobby for the nation’s “Command of the Sea,” and which boasted 15,000 members by the turn of the century.^113^ While it would contribute to the formation of the modern Sea Cadet Corps in 1919, its first foray into the training of boys occurred with the establishment of a school at Liscard, near Liverpool, in 1903. This formed part of a broader response to contemporary war scares which prompted continual demands for greater investment in both the Royal and Merchant Navies. The Navy League’s Training School followed the GHS lead in admitting only boys of “good physique”—although as a charitable endeavor they actively recruited amongst the urban poor. The League’s *Journal* presented before-and-after photographs of dirty, destitute boys being transformed into promising future sailors with the help of a wash, haircut, and uniform.^114^ Such changes were purely cosmetic; the boys needed to meet minimal qualifying measurements in order to be admitted. As the Liscard School’s founder, George C. Thomas, insisted, there was no place for “weaklings” in his institution.^115^ Instead, he argued that “pinched, undersized, and undernourished boys of no physique” had no prospect of becoming robust British seamen, and declared that they should not “be trained for a service that, form its very nature, they are not physically adapted for.”^116^ Such sentiments offered a stern rebuke to the composition of industrial training ships, with their complements of undersized, impoverished boys. Fitness for the sea was not thought to be universal, nor was it thought to be possible to raise any poor boy to the desired standard.

Thomas was a former captain of the mercantile marine, so it was perhaps inevitable that far more of the Liscard boys ended up in the merchant service as the Navy.^117^ Certainly, the Navy League was overt in its desire to mold an entirely new generation of healthy British seafarers capable of ridding the commercial fleet of foreign influence, and used their fundraising materials to deride the 40,000 foreign seamen in the service who were cumulatively pocketing two million pounds a year in wages.^118^ Unlike the Navy, which was dismissive of poor boys given their “unfortunate position in life,” the Navy League believed that such individuals could be trained into effective replacements for the “aliens” operating British vessels. After all, they noted, if Nelson had been able to muster a successful force for Trafalgar from “corner boys and ragamuffins,” then it should be possible to furnish a new generation of “heroes of the streets.”^119^

The Navy League’s inclusive and patriotic messaging was somewhat undermined by the stiff criteria they put into place to ensure that only strong and healthy boys, with superior eyesight and dentition, be allowed into the school. It is true that the measurements they set were slightly below that of the Royal Navy, but they were only receptive to children of impoverished backgrounds as long as their physiques showed no signs of material deprivation.^120^ The Training School did not, however, dispense with the improving effects of nurture, and placed a considerable emphasis on diet and exercise to help hone the boys’ muscular development. Still, the Navy League’s recruitment criteria suggested that modern “ragamuffins” would be unlikely to qualify for either the school or a future association with the Royal Navy or Naval Reserve. While British commercial vessels rarely insisted on any particular physical standards for their crews, those wishing to qualify as naval reservists had to exhibit the same minimum measurements as ordinary naval recruits. Given that the Training School at Liscard was explicitly funneling boys in this direction, it followed the same premises motivating the GHS adoption of metrical barriers for admission. While both schools actively sought to improve the physiques of their charges over the course of their training, significant shortfalls in height or chest circumference could not necessarily be remedied even with intensive effort between the ages of 13 and 15. Smaller boys did not seem worth the investment when their capacity for growth was unreliable.

The Marine Society, which had no expectation of preparing boys for the Naval Reserve, arguably demonstrated a greater commitment to philanthropy in its willingness to train undersized applicants. From the Navy League’s perspective, the Marine Society’s success in transforming destitute children into viable sailors was no less than a national service.^121^ Strong, healthy candidates were always preferred aboard their training ship *Warspite*, but regular drill, gymnastics, marching, and swimming practice exerted a positive influence on even the most “poor and distressed” boys. Indeed, the Marine Society’s training clearly demonstrated the beneficial effects of a nourishing diet and healthy exercise on children’s physical development. They may have avoided qualifying measurements, but anthropometric evidence validated their approach: boys who were short and under-developed on joining the *Warspite* finished above average in weight, girth, and height when leaving.^122^

The expansion of maritime training programmes around the turn of the century offered a variety of models for producing healthier boy sailors for both of Britain’s sea services. While the diffuse state of the mercantile marine—with its wide assortment of owners and operators—ensured that physical standards were never widely implemented, contemporary accusations of merchant sailors’ degeneracy still provoked considerable agitation amongst a certain breed of nationalist commentator. Their concerns equally encouraged the training of British boy sailors in order to enlarge the Naval Reserve and guarantee the loyalties of merchant crews during wartime. While state-run industrial and reformatory ships failed to produce many candidates for the merchant service, charitable organizations such as the Navy League and Marine Society successfully emphasized the quality as well as quantity of their recruits. Following the Navy’s lead, they encouraged boys’ growth through improved victualling and targeted exercises, and employed measurements to track their development—showing that even underprivileged children could be molded into healthy adults. While this improved generation of British sailors may have been the result of scaremongering rather than a genuine manning crisis in the merchant sector, their presence demonstrated that a concentrated investment in the health and welfare of young citizens could lift the nation’s prospects.

## Conclusion

By the early twentieth century, there were approximately 7,000 British boys being trained for the sea by the Royal Navy, and another 2,000 by charitable organizations.^123^ The increased use of selective recruitment criteria and an emphasis placed on healthy growth and development not only led to praise of the boys’ physiques, but was also credited with raising the physical condition of the entire Navy. No longer were sailors “picked up anywhere and anyhow,” as the naval hygienist Walter Reid noted in 1900, but instead were examined “most carefully as a boy, and systematically educated and trained to the performance of the varied and arduous duties required of him.”^124^ The transformation of naval crews into an intelligent, adaptable, and fit fighting force had been accomplished after several decades of implementing health reforms directed at adult seafarers and boy recruits—both at the Greenwich Hospital School and throughout the Navy’s network of training ships. This transformative period coincided with a popular preoccupation with the threat of national degeneration and its prospective impact on Britain’s maritime fortunes. The Navy’s response to such fears was to adopt an approach that eschewed the emerging dichotomy between nature and nurture as means to improvement: on the one hand, they raised enlistment standards to favor well-nourished boys from prosperous backgrounds, and on the other, emphasized the cultivation of strength through diet, exercise, and temperance. The merchant service followed a similar path towards improvement, with charitable schemes increasingly dedicated to helping boys of good character develop the physical qualities associated with male athleticism.

As a result of these processes, the bodies of seafarers and trainees were under constant observation to determine whether they were improving or deteriorating in physique. Using anthropometric indicators, naval medical authorities increasingly measured the effects that sport, recreation, and dietary and drink reforms had on the male body. The Greenwich Hospital School in particular became a laboratory for testing methods of fostering physical development—demonstrating the positive and negative effects of regimen changes on its students’ health in the final decades of the nineteenth century. The Marine Society’s success in preparing economically disadvantaged recruits for the merchant service provided further evidence that lifestyle interventions at a young age could yield healthier adult bodies.

Ambivalence about the limits of such transformations nevertheless prevailed. Both the Royal Navy and Navy League explicitly targeted larger boys for their training schemes, and rejected any applicant who failed to meet the minimum physical standards they imposed. Indeed, the Navy had zealously promoted the use of height, weight and chest measurements as arbitrators of fitness throughout the nineteenth century, despite ongoing debates around adolescent growth patterns and the most accurate means of obtaining data. This insistence on qualifying measurements ultimately allowed for the disproportionate recruitment of candidates from more prosperous backgrounds, creating a force whose respectability was as much a matter of social class as it was a physically-embodied ideal. Had such machinations been clear to reformers championing environmental solutions to the specter of national decay, it is doubtful that the boys in training for the sea services would have been taken as a fair model to emulate.

## Footnotes

1 Jonas Hanway (1712-1786), founder of the Marine Society, made this point to justify the expansion of youth training initiatives. See Jonas Hanway, *Reasons for an Augmentation of at least Twelve Thousand Mariners* (London: R. and J. Dodsley, 1759), 92.

2 For an overview of boys’ participation in the Royal Navy in the age of sail, see Roland Pietsch, *The Real Jim Hawkins: Ships’ Boys in the Georgian Navy* (Barnsley: Seaforth, 2010) and D. A. B. Ronald, *Young Nelsons: Boy Sailors During the Napoleonic Wars* (Oxford: Osprey, 2012).

3 See e.g., Alice Broadman Smuts, *Science in the Service of Children, 1893-1935* (New Haven: Yale University Press, 2006); Roger Cooter, ed., *In the Name of the Child: Health and Welfare, 1880-*1940 (London: Routledge, 1992); Anna Davin, “Imperialism and Motherhood,” *History Workshop Journal* 5 (1978): 9-66; Deborah Dwork, *War is Good for Babies and Other Young Children* (London: Routledge, 1987); Thomas E. Jordan, *The Degeneracy Crisis and Victorian Youth* (Albany: SUNY Press, 1993); Hilary Marland, *Health and Girlhood in Britain, 1874-1920* (Basingstoke: Palgrave Macmillan, 2013).

4 See Michael Brown, “Cold Steel, Weak Flesh: Mechanism, Masculinity and the Anxieties of Late Victorian Empire,” *Cultural and Social History* 14 (2017): 155-181; Michael Anton Budd, *The Sculpture Machine: Physical Culture and Body Politics in the Age of Empire* (Basingstoke: Palgrave Macmillan, 1997); Bruce Haley, *The Healthy Body and Victorian Culture* (Cambridge, MA: Harvard University Press, 1978).

5 On fin-de-siècle degeneration theory, see J. Edward Chamberlin and Sander L. Gilman, *Degeneration: The Dark Side of Progress* (New York: Columbia University Press, 1985); Daniel Pick, *Faces of Degeneration: A European Disorder, c.1848-1918* (Cambridge: Cambridge University Press, 1989).

6 Heinrich Hartmann, *The Body Populace: Military Statistics and Demography in Europe before the First World War* (Cambridge, MA: MIT Press, 2019).

7 Dr. Robert Hutchison, in *Minutes of Evidence Taken Before the Inter-Departmental Committee on Physical Deterioration, Vol. II*. (London: His Majesty’s Stationary Office, 1904), 373.

8 On the role played by the British Army in fueling anxieties about degeneration, see Richard Soloway, “Counting the Degenerates: The Statistics of Race Deterioration in Edwardian England,” *Journal of Contemporary History* 17 (1982): 137-164, and Vanessa Heggie, “Lies, Damn Lies, and Manchester’s Recruiting Statistics: Degeneration as an ‘Urban Legend’ in Victorian and Edwardian Britain,” *Journal of the History of Medicine and Allied Sciences* 63 (2008): 178-216.

9 Mary A. Conley, *From Jack Tar to Union Jack: Representing Naval Manhood in the British Empire, 1870-1918* (Manchester: Manchester University Press, 2009).

10 See the propagandistic stories of the late-Victorian Navy such as Edmund R. Freemantle, “The British Bluejacket,” *The Navy and Army Illustrated* 1 (3 January 1896): 29-30.

11 Oliver Walter, “‘A Great Improvement in the Sailor’s Feeling towards the Naval Service’: Recruiting Seamen for the Royal Navy, 1815-1853,” *Journal for Maritime Research* 12 (2010): 27-57.

12 An overview of these reforms can be found in Eugene Rasor, *Reform in the Royal Navy: A Social History of the Lower Deck, 1850-1880* (Hamden, CT: Archon, 1976).

13 See for example Richard Blake, *Religion in the British Navy, 1815-1879: Piety and Professionalism* (Woodbridge: Boydell Press, 2014); Conley, *From Jack Tar to Union Jack*; Isaac Land, *War, Nationalism, and the British Sailor, 1750-1850* (Basingstoke: Palgrave Macmillan, 2009); Michael Paris, *Warrior Nation: Images of War in British Popular Culture, 1850-2000* (London: Reaktion, 2000).

14
*Regulations and Instructions for the Medical Officers of His Majesty’s Fleet* (London: W. Clowes and Sons, 1835), 38.

15 Alexander Armstrong, *Observations of Naval Hygiene and Scurvy* (London: John Churchill, 1858), 83.

16 One of the earliest anthropometric studies commissioned in Britain surveyed the size of children employed in factories. *First Report of His Majesty’s Commissioners on Employment of Children in Factories* (London: House of Commons, 1833).

17 John Wilson, *Outlines of Naval Surgery* (Edinburgh: MacLachlan, Stewart & Co, 1846), 4, 8.

18 See e.g., Frederick James Brown, *Questions and Observations in Hygiene: Recommended to the Consideration of Naval Medical Men* (London: John Churchill, 1849), 63-64; Armstrong, *Naval Hygiene*, 80-84.

19
*The Queen’s Regulations and the Admiralty Instructions for the Government of Her Majesty’s Naval Service* (London: Her Majesty’s Stationary Office, 1862), 398.

20 Wilson, *Naval Surgery*, 33. For an overview of naval hygiene reforms in the nineteenth century, see Elise Juzda Smith, “‘Cleanse or Die’: British Naval Hygiene in the Age of Steam, 1840-1900,” *Medical History* 62 (2018): 177-198.

21 Gilbert Blane, *A Brief Statement of the Progressive Improvement of the Health of the Royal Navy* (London: W. Nicol, 1830), 29; Alexander Bryson, *Report on the Climate and Principal Disease of the African Station* (London: William Clowes and Sons, 1847), 9.

22 W. F. Bynum, “Alcoholism and Degeneration in 19^th^-Century European Medicine and Psychiatry,” *Addiction* 79 (1984): 59-70; Heidi Rimke and Alan Hunt, “From Sinners to Degenerates: The Medicalisation of Morality in the Nineteenth Century,” *History of the Human Sciences* 15 (2002): 59-88.

23 Judith R. Walkowitz, *Prostitution and Victorian Society: Women, Class, and the State* (Cambridge: Cambridge University Press, 1980), 67-148.

24
*Statistical Report of the Health of the Royal Navy, For the Year 1863* (London: House of Commons, 1866), iii-iv.

25 WH Sloggett of the *Edgar*, in *Statistical Report of the Health of the Royal Navy, For the Year 1864* (London: House of Commons, 1867), 16.

26 F.F. Sankey, *Familiar Instructions in Medicine and Surgery with Observations on the Means of Maintaining the Health of Men on Ship Board* (London: Parker, Furnivall, and Parker, 1846), 30.

27 John D. MacDonald, *Outlines of Naval Hygiene* (London: Smith, Elder, & Co, 1881), 176.

28 “The Winter Season,” *In Training* 1 (1898): 35-36.

29 Bryson, *African Station*, 209.

30
*Report of the Committee to Inquire into the Expediency of Reducing the Present Quantity of Spirits Served out Daily to the Seamen in the Royal Navy* (London: Her Majesty’s Stationary Office, 1850).

31 S. G. Wintz, *Temperance Work in the Royal Navy* (London: Hodder and Stoughton, 1879), 1-2.

32 C. March Beadnell*, Physical Measurements of the Sailor* (London: John Bale, Sons & Danielsson, 1901), 3-4.

33 Alfred Jephson, “Men-of-War’s Men,” *The Navy and Army Illustrated* 1 (6 March 1896): 126.

34 The Exhibition attracted more than two million visitors between 2 May and 24 October 1891. See Pieter van der Merwe, “Views of the Royal Naval Exhibition, 1891,” *Journal for Maritime Research* 3 (2001): 146-156.

35 “Gymnastics in the Navy,” *The Navy and Army Illustrated* 4 (17 September 1897): 264.

36 See e.g., Charles Beresford, “Some of the Naval Needs of the Nation,” *The Navy and Army Illustrated* 4 (1897): 85-86.

37
*Royal Commission on Unseaworthy Ships: Vol II: The Minutes of Evidence and the Appendix* (London: Her Majesty’s Stationary Office, 1874).

38 Brown, *Questions and Observations in Hygiene*, 63.

39 “Notices,” *The Standard* (6 January 1882), 5.

40 “Boying the Navy,” *Judy* (18 January 1882), 34.

41 David McLean, *Education and Empire: Naval Tradition and England’s Elite Schooling* (London: British Academic Press, 1998).

42 The titles Lower and Upper School were formally adopted in 1825, and were collectively known as the Royal Hospital Schools. In 1892, the name was officially changed to the Greenwich Royal Hospital School. H.D. Turner, *The Cradle of the Navy: The Story of the Royal Hospital School at Greenwich and at Holbrook, 1694-1988* (York: William Sessions Ltd, 1990), 41.

43 Ibid.

44 “Qualifications for the Upper School of the Royal Hospital, Greenwich,” Undated 1850s, ADM 73/221/1, Admiralty Records, The National Archives, London.

45 See for example, Greenwich Hospital School to Louisa Jackson, 5 May 1869, ADM 66/79, Admiralty Records, The National Archives, London.

46 Admissions Form to Upper School of Greenwich Hospital, 1855, ADM 73/221/6, Admiralty Records, The National Archives, London.

47 “Rules and Regulations for the Admission of Children into the Lower School of this Institution,” Undated 1860s, ADM 73/221/2, Admiralty Records, The National Archives, London.

48 “Circular No. 63,” 20 August 1831, ADM 7/889, Admiralty Records, The National Archives, London.

49 Factory surveys from Manchester, Stockport, and Leeds, undertaken in 1833 and 1837, showed 14-year-old boys averaged between 4’7” to 4’8” in height. See J. M. Tanner, *A History of the Study of Human Growth* (Cambridge: Cambridge University Press, 1981), 147-161.

50 A height standard for 17, 18, and 19 year old recruits was added at the same time, although weights were not specified for these groups. “Memorandum,” 25 May 1841, ADM7/889, Admiralty Records, The National Archives, London.

51 “Circular No.74**:** Regulations Respecting Naval Apprentices & Boys,” 20 February 1851, ADM7/890, Admiralty Records, The National Archives, London.

52
*Copies of a Correspondence Between the Board of Treasury and The Board of Admiralty, on the Subject of the Manning of the Royal Navy*, *Together with Copies of a Report of a Committee of Naval Officers and of Her Majesty’s Order in Council Relating Thereto* (London: Her Majesty’s Stationer, 1853), 13.

53 See for example, *Royal Commission on Employment of Children in Factorie*s, and Adolphe Quetelet, “Recherches sur la loi de la croissance de l'homme,” *Nouveaux mémoires de l'Académie Royale des Sciences et Belles-Lettres de Bruxelles* 7 (1832): 1-32.

54
*Copies or Extracts Of the Report and Appendix of the Committee of 1852 on Manning the Navy* (London: House of Commons, 1859), 10.

55 Ibid., 11.

56 William Aitken, *On the Growth of the Recruit and Young Soldier* (London: Griffin, Bohn, 1862), 19-20.

57 “Instructions for the Medical Examination of Boys selected for admission to Greenwich Hospital School at 13 Years of Age,” 13 July 1871, ADM 169/83, Admiralty Records, The National Archives, London.

58 “Circular No.30: Examination of Second Class Boys on Entering the Navy,” 30 April 1873, ADM7/893, Admiralty Records, The National Archives, London.

59 “Admiralty to the Medical Director General of the Admiralty, 21 July 1875, ADM 169/83, Admiralty Records, The National Archives, London.

60 Walter Reid to Sir Henry Keppel, 29 April 1875, ADM169/83, Admiralty Records, The National Archives, London.

61 Charles Burney to Admiralty, 7 May 1875, ADM 169/83, Admiralty Records, The National Archives, London.

62 G. W. Armstrong to Charles Burney, 6 May 1875, ADM 169/83, Admiralty Records, The National Archives, London.

63 Roberta Bivins and Hilary Marland, “Weighing for Health: Management, Measurement, and Surveillance in the Modern Household,” *Social History of Medicine* 29 (2016): 768.

64 See e.g., the testimony of Captain Robert Harris in *Report of the Commissioners on Manning the Navy* (London: Her Majesty’s Stationery Office, 1859), 259. The chest standard for second-class boys entering the Navy was introduced in “Circular No. 30: Examination of Second Class Boys on Entering the Navy,” ADM7/893, Admiralty Records, The National Archives, London.

65 For a discussion on the lack of standards in recording chest measurements, see Hartmann, *The Body Populace*, 72-77.

66 A Naval Officer, “The Bluejackets and Marines of the Royal Navy,” *Fraser’s Magazine* 12 (1875): 142.

67 Superintendent, GHS to Captain Hood, 4 February 1869, ADM 66/79, Admiralty Records, The National Archives, London.

68 Superintendent, GHS to Captain Stirling, 9 April 1869, ADM 66/79, Admiralty Records, The National Archives, London.

69
*Report of the Committee Appointed by the Lords Commissioners of the Admiralty to Inquire into Greenwich Hospital School* (London: Her Majesty’s Stationer, 1882), 20.

70
*Report of the Committee Appointed to inquire into the present Condition, Cost, and Utility of Greenwich Hospital School* (London: House of Commons, 1870), 9.

71 Turner, *Cradle of the Navy*, 75-76.

72
*Report of the Committee Appointed by the Lords Commissioners of the Admiralty to Inquire into Greenwich Hospital School*, xiii.

73 Ibid., xii-xiii, xvi, 98.

74 “Greenwich Hospital School,” *The Lancet* 119 (17 June 1882): 1007.

75 Charles Burney to Admiralty, 17 June 1882, ADM 169/118, Admiralty Records, The National Archives, London.

76 Fleet Surgeon Henry Hadlow to Admiralty, 2 November 1882, ADM 169/118, Admiralty Records, The National Archives, London.

77 Yuriko Akiyama, *Feeding the Nation: Nutrition and Health in Britain Before World War One* (London: Taurus, 2008), 163-165.

78 Report of the Committee of Inspection to Greenwich Hospital School, 7 July 1884, ADM 203/02, Admiralty Records, The National Archives, London.

79 Ibid.

80 Ibid.

81 Report of the Committee of Inspection to Greenwich Hospital School, 20 July 1885, ADM 203/02, Admiralty Records, The National Archives, London.

82 Robert Holme to Admiralty, 26 April 1864, ADM 65/58, Admiralty Records, The National Archives, London.

83 “Greenwich Hospital School, Part II,” *The Navy and Army Illustrated* 6 (10 September 1898): 600.

84 Francis H. Miller, “The Food of the Bluejacket,” *The Navy and Army Illustrated* 2 (4 September 1896): 110.

85 Naval Officer, “Bluejackets,” 139.

86 “The Royal Naval School, Greenwich,” *Illustrated London News* (6 April 1889), 437.

87 Various iterations of this vessel, called *Fame*, were used at the Greenwich Hospital Schools between the 1840s and 1950s, and the Navy’s training ships were also decommissioned sailing vessels. See Turner, *Cradle of the Navy*, 51-54.

88 “Making Men-of-War’s Men,” *The Navy and Army Illustrated* 4 (11 June 1897): 26.

89 For a discussion of the rise of the physical culture movement, see Ina Zweiniger-Bargielowska, *Managing the Body: Beauty, Health, and Fitness in Britain, 1880-1939* (Oxford: Oxford University Press, 2013), 17-61.

90
*The Principles and Practice of Educational Gymnastics for the Use of Officers and Physical Training Instruction in His Majesty’s Fleet* (London: His Majesty’s Stationary Office, 1908), 6.

91 For perspectives on the militarized athleticism promoted in British schools, see David Kirk, *Schooling Bodies: School Practice and Public Discourse, 1880-1950* (London: Leicester University Press, 1998); Alan Penn, *Targeting Schools: Drill, Militarism and Imperialism* (London: Woburn Press, 1999); J. A. Mangan, *Athleticism in the Victorian and Edwardian Public School* (Cambridge: Cambridge University Press, 1981).

92 See e.g., “Making Men-of-War’s Men,” 27.

93 Walter Vernon Anson to Admiralty, 4 February 1902, ADM 203/02, Admiralty Records, The National Archives, London.

94 Lionel Rose, *The Erosion of Childhood: Child Oppression in Britain, 1860-1918* (London: Routledge, 1991), 145-160.

95 Henry Hadlow, “Short Sight Amongst the Boys of Greenwich Hospital School,” *British Medical Journal* 1:1168 (19 May 1883): 952-954.

96 “Anthropometry at Eton,” *The Times* (26 December 1907), 2.

97 Superintendent, GHS to Admiralty, 15 October 1895, ADM 169/200, Admiralty Records, The National Archives, London.

98 A Naval Officer, “Bluejackets and Marines,” 142.

99 For a contemporary economic justification for the exact physical standards set by the Navy, see Archibald S. Hurd, “Boys for the Navy: How Bluejackets are Trained,” *Young England* (Undated 1880s), 297.

100 Gordon Stables, “How to Become an Apprentice or Midshipman in the Merchant Service,” *Boys Own Paper* (9 November 1889), 94.

101 “The Lads of the Training Ship ‘Impregnable,’” *The Navy and Army Illustrated* 2 (24 July 1896): 43.

102 George C. Thomas, *The Training of Boys for the Sea Service and Recruiting for the Royal Navy* (Liverpool: George L Miller & Co, 1905), 5.

103 For Victorian medical perceptions of merchant seafarers, see Tim Carter, *Merchant Seamen’s Health, 1860-1960* (Woodbridge: Boydell, 2014), 23-50.

104 Royal Commission on Unseaworthy Ships, *Final Report of the Commissioners, Minutes of the Evidence, and Appendix* (London: George Edward Eyre and William Spottiswoode, 1874).

105 Charles Griffiths, *Numerical Decline of British Seamen* (Birmingham: Moody Bros, 1876), 3.

106 “The Lancashire Navy Sea Training Home for Poor Boys,” *The Navy League Journal* 10 (1905): unpaginated insert.

107 M. F. Crisp, *Letter to British Shipowners, etc. on Manning the Navy; Portraying the Characters of Seamen, and Exhibiting their Power and Value in our Colonial Empire* (Calcutta: Printer for the Publisher, 1846), iv.

108 A Captain, RN, *Hints on Educating and Manning the Navy* (Bath: Hayward & Payne, 1857), 7.

109 Barry Godfrey, Pamela Cox, Heather Shore, and Zoe Alker, *Young Criminal Lives: Life Course and Life Chances from 1850* (Oxford: Oxford University Press, 2017), 86-110.

110 See e.g., “Octopus,” *How Ships Are Lost, And Why So Difficult to Man Them With Seamen* (London: J. D. Potter, 1875).

111 Thomas Brassey, *British Seamen as Described in Recent Parliamentary and Official Documents* (London: Longman, Greens, and Co.), 69.

112 Brassey’s views on recruitment were debated at the Royal United Service Institution in 1876, and several respondents, including Edwin Chadwick, defended the practice of training poorer boys. See Thomas Brassey, *How Best to Improve and Keep Up the Seamen of the Country* (London: Harrison and Sons, 1876), 15-41.

113 For an early history of this organisation, see Matthew Johnson, “The Liberal Party and the Navy League in Britain before the Great War,” *Twentieth Century British History* 22 (2011): 137-163.

114 “Navy League Sea Training Home at Liscard,” *Navy League Journal* 9 (1904): 243-245.

115 Thomas, *Training of Boys*, 18.

116 Ibid.

117 In 1905, the school sent 52 boys to the mercantile marine, and only 6 to the Royal Navy. “The Lancashire Navy League Sea-Training Home,” *Navy League Journal* 10 (1905): 322.

118 Henry T. C. Knox, “The Lancashire Navy League Sea-Training Home,” *Navy League Journal* 10 (1905): X.

119 “Home for Poor Boys,” unpaginated insert.

120 In 1905, 15-year-olds at the school had to be 5’1” tall with a 30-31” chest, compared with the Navy’s requirement of 5’2” in height with a 33” chest. Ibid.

121 “The Training Ship *Warspite*,” *Navy League Journal* 9 (1904): 221.

122 For a table of the *Warpsite* boys’ changing measurements over a 12-month period, see *Minutes of Evidence Taken Before the Departmental Committee on the Supply and Training of British Boy Seamen for the Mercantile Marine* (London: His Majesty’s Stationer, 1908), 401.

123
*Report of the Committee Appointed by the Board of Trade to Inquire into the Supply and Training of British Boy Seamen for the Mercantile Marine* (London: His Majesty’s Stationer, 1908), 9.

124 Walter Reid, “The Duties of Naval Surgeons,” *British Medical Journal* 1:1473 (23 March 1889): 645.

